# Determinants of cancer incidence and mortality among people with vitamin D deficiency: an epidemiology study using a real-world population database

**DOI:** 10.3389/fnut.2023.1294066

**Published:** 2023-12-07

**Authors:** Yi-Chen Lai, Yu-Han Chen, Fu-Wen Liang, Yu-Cih Wu, Jhi-Joung Wang, Sher-Wei Lim, Chung-Han Ho

**Affiliations:** ^1^Department of Emergency Medicine, An Nan Hospital, China Medical University, Tainan, Taiwan; ^2^Department of Family Medicine, An Nan Hospital, China Medical University, Tainan, Taiwan; ^3^Department of Public Health, College of Health Sciences, Kaohsiung Medical University, Kaohsiung, Taiwan; ^4^Department of Medical Research, Kaohsiung Medical University Hospital, Kaohsiung, Taiwan; ^5^Center for Big Data Research, Kaohsiung Medical University, Kaohsiung, Taiwan; ^6^Department of Medical Research, Chi-Mei Medical Center, Tainan, Taiwan; ^7^Department of Anesthesiology, Tri-Service General Hospital and National Defense Medical Center, Taipei, Taiwan; ^8^Department of Neurosurgery, Chi Mei Medical Center, Chiali, Tainan, Taiwan; ^9^Department of Nursing, Min-Hwei College of Health Care Management, Tainan, Taiwan; ^10^Department of Information Management, Southern Taiwan University of Science and Technology, Tainan, Taiwan; ^11^Cancer Center, Taipei Municipal Wanfang Hospital, Taipei Medical University, Taipei, Taiwan

**Keywords:** vitamin D deficiency, cancer incidence, mortality risk, Taiwan cancer registry, real-world data

## Abstract

**Introduction:**

This study aimed to investigate the determinants of cancer incidence and mortality in patients with vitamin D deficiency using a real-world population database.

**Methods:**

We utilized the International Diagnostic Classification Code (ICD9:268 / ICD10: E55) to define patients with vitamin D deficiency. Additionally, the Cox regression model was used to estimate overall mortality and identify potential factors contributing to mortality in cancer patients.

**Results:**

In 5242 patients with vitamin D deficiency, the development of new-onset cancer was 229 (4.37%) patients. Colon cancer was the most prevalent cancer type. After considering confounding factors, patients aged 50–65 and more than 65 indicated a 3.10-fold (95% C.I.: 2.12–4.51) and 4.55-fold (95% C.I.: 3.03–6.82) cancer incidence, respectively compared with those aged <50. Moreover, patients with comorbidities of diabetes mellitus (DM) (HR: 1.56; 95% C.I.: 1.01–2.41) and liver disease (HR: 1.62; 95% C.I.: 1.03–2.54) presented a higher cancer incidence rate than those without DM/ liver disease. In addition, vitamin D deficiency patients with cancer and dementia histories indicated a significantly higher mortality risk (HR: 4.04; 95% C.I.: 1.05– 15.56) than those without dementia.

**Conclusion:**

In conclusion, our study revealed that vitamin D deficiency patients with liver disease had an increased incidence of cancer, while those with dementia had an increased mortality rate among cancer patients.

## 1 Introduction

Cancer is a leading cause of death, and the burden of cancer incidence and mortality is increasing rapidly worldwide. In light of this public health concern, it is imperative to identify adjunctive therapies that are not only effective but also safe for cancer prevention. For those affordable and innovative approaches to address this challenge, the association between vitamin D and the risk of cancer has been extensively investigated in recent decades (1). Vitamin D and its metabolites have been shown to have anti-cancer activity by multiple mechanisms, including the inhibition of tumor angiogenesis (2, 3), clonal proliferation (4, 5), and the promotion of cell differentiation and apoptosis (6, 7). Vitamin D deficiency, defined as a low serum level of 25(OH)D, has been found to be associated with a variety of cancer risks (8–10) and was also confirmed to be highly prevalent in people with different types of cancer (7). A meta-analysis of randomized controlled trials (RCTs) also demonstrated a moderate inverse association of circulating 25(OH)D concentration with total cancer incidence (11). However, clinical evidence regarding the association between vitamin D supplementation and a reduction in total cancer incidence remains inconclusive (12–14). Furthermore, a large cohort study conducted in Denmark, which included 217,244 individuals, revealed a significant association between higher vitamin D levels and an increased incidence of skin, prostate, and hematological cancers (15). These findings indicated a complex relationship between vitamin D levels and different cancer types.

In regard to cancer mortality, most of the previous literature demonstrated a reverse relationship between circulating 25(OH)D concentration and cancer mortality (11, 16). In a large cohort from the UK Biobank, involving 365,530 participants, higher 25(OH)D concentrations were associated with an 11% lower risk of cancer mortality, demonstrating a non-linear inverse association (17). The majority of systemic reviews and meta-analyses of RCTs also showed that the supplementation of vitamin D reduced total cancer death (13, 14, 18). However, it is important to note that the most recent meta-analysis, which included 12 RCTs with a total of 72,669 adults, reported contradictory findings. The results from this analysis indicated that vitamin D supplementation did not reduce cancer mortality risk (19). The D-Health Trial, a nationwide RCT conducted by Neale et al. in Australia, demonstrated that administering vitamin D monthly to unscreened older people did not reduce all-cause mortality but was associated with a trend of increased risk of death from cancer (20). The existing literature presents controversies and uncertainties regarding the impact of unscreened vitamin D supplementation on cancer incidence and mortality. This highlights the critical need to identify the high-risk group of individuals with vitamin D deficiency who are most likely to benefit from supplementation. However, there is a scarcity of studies specifically investigating the cancer risks associated with vitamin D deficiency.

To address the knowledge gaps and facilitate more targeted health education, as well as establish the potential benefits of vitamin D supplementation on the population of certain characteristics, this study aimed to conduct an epidemiology study to explore the potential risk of cancer incidence and mortality among patients with vitamin D deficiency. By understanding the relationship between vitamin D deficiency and cancer outcomes, we would like to provide valuable information that could improve health outcomes for patients at higher risk.

## 2 Materials and methods

### 2.1 Data sources

The National Health Insurance Research Database (NHIRD) and the Taiwan Cancer Registry (TCR) were used to select patients with vitamin D deficiency and the interesting outcome, cancer, in this study. NHIRD was an administrative claim from Taiwan’s National Health Insurance Program, which has been a single-payer insurance program since 1996 and covers almost 99% of the population of Taiwan (21). Taiwan’s National Health Insurance Program is a comprehensive healthcare program that includes all basic medical expenses, such as outpatient visits, inpatient care, prescription medications, traditional Chinese medicine treatments, dental services, surgical procedures, and diagnostic investigations (21). In addition, TCR has been a population-based cancer registry database with cancer-related information since 1979 and covers approximately 99% of all cancer cases in Taiwan. All hospitals in Taiwan are legally obligated to submit cancer data to TCR, providing a comprehensive record of their relevant information, including cancer patients’ demographics, primary cancer sites, tumor histology, and treatment types. For research purposes, Taiwan’s Health and Welfare Data Science Center (HWDC) integrated the different health-related datasets and eliminated identifying data to avoid violations of personal information protection. This study was conducted in compliance with the Declaration of Helsinki and approved by the Ethics Committee of the Institutional Review Board of Chi-Mei Hospital (IRB: 11012-E02). All methods were performed in accordance with relevant guidelines and regulations.

### 2.2 Study population

**Figure 1:**
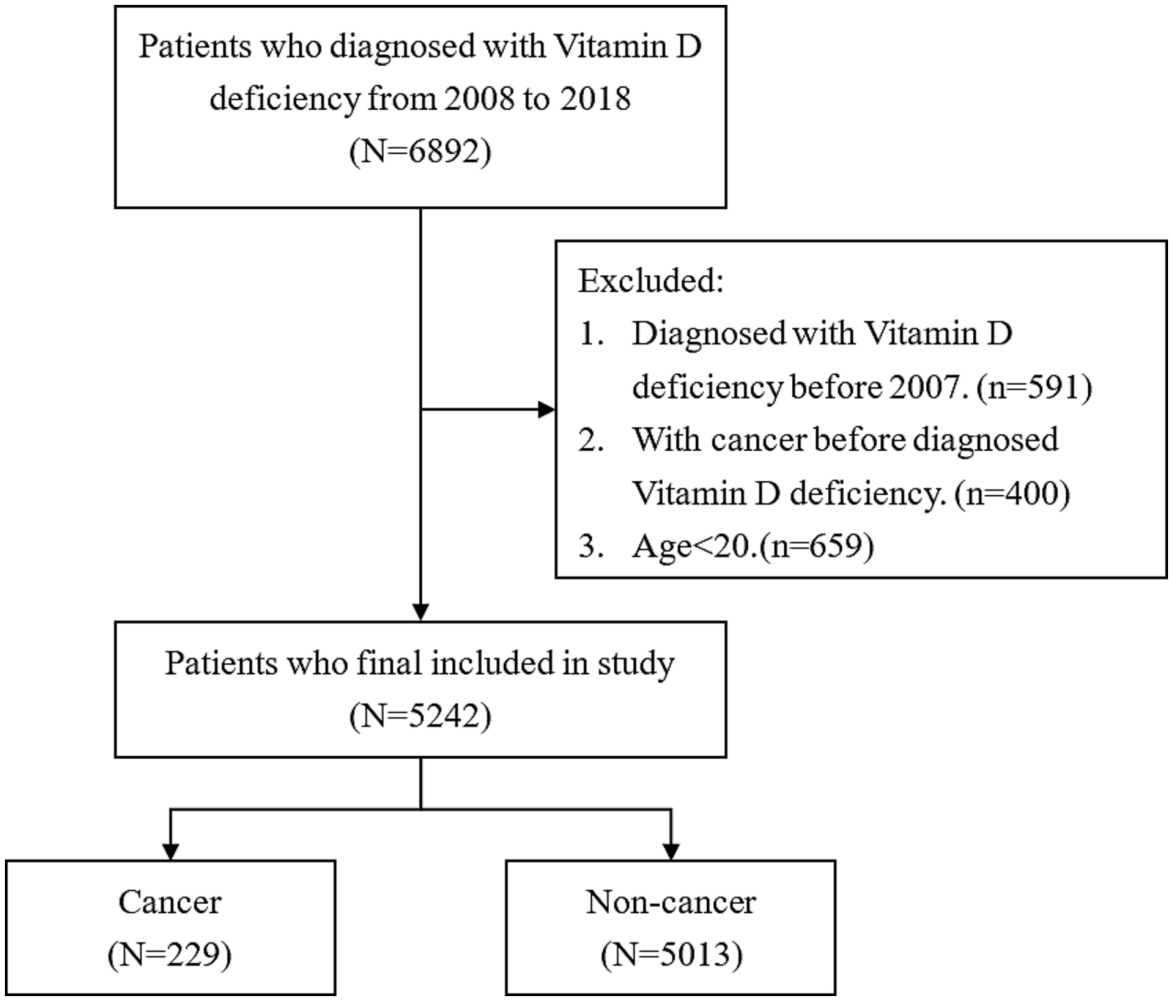
Flowchart of patients’ selection.

Patients with new-onset vitamin D deficiency and whose diagnosis age was more than 20 years old were selected from NHIRD between 2008 and 2018. The identification of vitamin D deficiency was carried out using the International Classification of Diseases, Ninth Revision, Clinical Modification (ICD-9-CM) code 268 from 2008 to 2015 or the International Classification of Diseases, Tenth Revision, Clinical Modification (ICD-10-CM) code E55 from 2016 to 2018. In addition, the aim of this study is to estimate cancer incidence. Therefore, patients with cancer history prior to the diagnosis date of vitamin D deficiency were excluded to avoid potential confounding bias in the estimation of cancer incidence. After the exclusion of patients aged less than 20 years and those with cancer history before being diagnosed with vitamin D deficiency, the remaining 5,242 patients were enrolled in this study. Figure 1 shows the flowchart of the selection process of patients.

In Taiwan, the coding of diagnosis codes, including vitamin D deficiency, is certified by the Bureau of National Health Insurance to ensure accuracy and prevent incorrect claims. Therefore, physicians apply diagnosis codes for vitamin D deficiency, which require supporting laboratory test results to confirm the condition. Generally, patients with a level of plasma 25(OH)D that is lower than 20 ng/mL (50 nmol/L) are classified as having vitamin D deficiency. This cut point was based on recognized guidelines and clinical standards. The process assured the accuracy and reliability of the diagnosis codes for vitamin D deficiency.

### 2.3 Outcome and measurements

The major outcome of this study was new-onset cancer based on the TCR using the International Classification of Diseases for Oncology, Third Edition (ICD-O-3). Patients with different cancer types were also presented. After the diagnosis date of vitamin D deficiency, all patients were followed until the diagnosis of cancer or censoring at death or the end date of the study, 31 December 2018. In addition, the secondary outcome would be to estimate the overall and cancer-specific mortality among patients with both vitamin D deficiency and cancer. The overall and cancer-specific mortality were identified as death due to any cause and cancer, respectively, from Taiwan’s cause of death dataset.

The other measurements included age, sex, the Charlson comorbidity index (CCI) score, and comorbidities. Age was classified into three groups for the purpose of analysis: <50 years, 50 to 65 years, and 65 years or older. This categorization allowed for the examination of age-specific patterns and associations in relation to cancer incidence among patients with vitamin D deficiency. CCI score was used to grade the severity of comorbid conditions. The CCI score was initially developed in 1984 through the review of hospital charts to evaluate the risk of 1-year mortality. The CCI assigned a weighting score to each diagnosis from a list of 19 medical conditions, with each comorbid condition ranging from 1 to 6 points, and the overall index was calculated by the sum of these scores. The CCI score has become a popular and widely used clinical index across various medical conditions and mortality. The application of the CCI score has provided evidence of its effectiveness in assessing comorbidity burden.

In addition, the selected comorbidities of this study were identified using the ICD-9-CM or ICD-10-CM based on the 1-year medical records prior to the diagnosis date of vitamin D deficiency. Those comorbidities included congestive heart failure (ICD-9-CM: 428, 425.4–425.9, 398.91, 402.01, 402.11, 402.91, 404.01, 404.03, 404.11, 404.13, 404.91, 404.93; ICD-10-CM: I09.9, I11.0, I13.0, I13.2, I25.5, I42.0, I425–I429, I43, I50, P29.0), cerebrovascular disease (ICD-9-CM: 430–438, 362.34; ICD-10-CM: G45, G46, I60–I69, H34.0, 290, 294.1, 331.2; ICD-10-CM: F00–F03, F05.1, G30, G31.1), dementia (ICD-9-CM: 290, 294.1, 331.2; ICD-10-CM: F00–F03, F05.1, G30, G31.1), chronic pulmonary disease (ICD-9-CM: 490–505, 416.8, 416.9, 506.4, 508.1, 508.8; ICD-10-CM: J40–J47, J60–J67, J68.4, J70.1, J70.3, I27.8, I27.9), renal disease (ICD-9-CM: 403.01, 403.11, 403.91, 404.02, 404.03, 404.12, 404.13, 404.92, 404.93, 582, 583.0–583.7, 585, 586, 588.0; ICD-10-CM: I12.0, I13.1, N03.2–N03.7, N05.2–N05.7, N18, N19, N25.0, Z49.0–Z49.2, Z94.0, Z99.2), liver disease (ICD-9-CM: 070.6, 070.9, 070.22, 070.23, 070.32, 070.33, 070.44, 070.54, 456.0–456.2, 570, 571, 572.2–572.8, 573.3, 573.4, 573.8, 573.9; ICD-10-CM: B18, I85.0, I85.9, I86.4, I98.2, K70.0–K70.4, K70.9, K71.1, K71.3–K71.5, K71.7, K72.1, K72.9, K73, K74, K76.0, K76.2–K76.9, Z94.4), diabetes mellitus (ICD-9-CM: 250; ICD-10-CM: E08–E13), hyperlipidemia (ICD-9-CM: 272; ICD-10-CM: E78), and hypertension (ICD-9-CM: 401–405; ICD-10-CM: I10-I13, I15).

### 2.4 Statistical analysis

The baseline characteristics of patients with vitamin D deficiency, such as age groups, sex, level of CCI score, and comorbidities, are presented as frequency with percentage. Pearson’s chi-square tests were used to evaluate the differences in distributions of categorical variables among patients with vitamin D deficiency between those with cancer and those without. The time to cancer among patients with vitamin D deficiency during the study period was presented as mean with standard deviation. Multivariable Cox proportional regressions were used to identify independent factors, including age, sex, level of CCI score, and comorbidities, associated with the risk of cancer incidence. The independent factors of overall and cancer-specific mortality were also estimated among patients with vitamin D deficiency and cancer. The cancer incidence and mortality risk were both estimated and reported as hazard ratios (HRs) with a 95% confidence interval (CI). All statistical analyses were conducted using SAS software, version 9.4 (SAS Institute Inc., Cary, NC, United States), and statistical tests were performed at a two-tailed significance level of 0.05.

## 3 Results

Of all 5,242 patients with vitamin D deficiency, the development of new-onset cancer was seen in 229 (4.37%) patients. Patients with vitamin D deficiency who were older than 65 years had a higher new-onset cancer rate (7.74%) than those aged between 50 and 65 years (5.35%) and those younger than 50 (1.88%). In addition, patients with vitamin D deficiency had a significantly higher cancer incidence rate when the CCI score was more than three (8.03%, *p* < 0.0001) compared with patients with a CCI score of 0 (3.18%) and those with a CCI score of 1 or 2 (6.26%). For all selected comorbidities, patients with vitamin D deficiency had the same new-onset cancer rate as those with a history of congestive heart failure (8.33%), cerebrovascular disease (6.01%), dementia (8.97%), chronic pulmonary disease (8.23%), renal disease (7.24%), liver disease (7.93%), diabetes mellitus (8.10%), hyperlipidemia (5.55%), and hypertension (6.41%). In all study subjects, the number of deaths was 402, while 19.4% of those were cancer patients. The results also indicated that the mean time to develop cancer among the patients with vitamin D deficiency was 3.06 ± 2.41 years (Table 1).

**Table 1:** Character of patients with vitamin D deficiency between those with and without cancer.

	Vitamin D deficiency (*N* = 5,242)	Non-cancer (*N* = 5,013)	Cancer (*N* = 229)	*p*-value
Sex, *n* (%)				
Male	1,461	1,390 (95.14)	71 (4.86)	0.2795
Female	3,781	3,623 (95.82)	158 (4.18)	
Age group, *n* (%)				
<50	2,339	2,295 (98.12)	44 (1.88)	<0.0001
50–65	1,663	1,574 (94.65)	89 (5.35)	
> = 65	1,240	1,144 (92.26)	96 (7.74)	
CCI group, *n* (%)				
0	3,457	3,347 (96.82)	110 (3.18)	<0.0001
1–2	1,374	1,288 (93.74)	86 (6.26)	
> = 3	411	378 (91.97)	33 (8.03)	
Comorbidity, *n* (%)				
Congestive heart failure	132	121 (91.67)	11 (8.33)	0.0240
Cerebrovascular disease	233	219 (93.99)	14 (6.01)	0.2102
Dementia	78	71 (91.03)	7 (8.97)	0.0450
Chronic pulmonary disease	316	290 (91.77)	26 (8.23)	0.0005
Renal disease	290	269 (92.76)	21 (7.24)	0.0138
Liver disease	391	360 (92.07)	31 (7.93)	0.0003
Diabetes mellitus	580	533 (91.9)	47 (8.1)	<0.0001
Hyperlipidemia	721	681 (94.45)	40 (5.55)	0.0953
Hypertension	1,232	1,153 (93.59)	79 (6.41)	<0.0001
Death, *n* (%)	402	324 (80.6)	78 (19.4)	<0.0001
Time to cancer, mean ± SD			3.06 ± 2.41	

Table 2 illustrates the development of cancer types among patients with vitamin D deficiency. This displayed the prevalence of various cancers in this specific population. The major top 10 cancer types were colorectal (32/229, 13.97%), followed by liver (31/229, 13.54%), breast (29/229, 12.66%), lung (25/229, 10.92%), hematopoietic system (19/229, 8.29%), thyroid (14/229, 6.11%), cervical, ovarian, and uterine (11/229, 4.80%), stomach (10/229, 4.37%), skin (9/229, 3.93%), and pancreatic (7/229, 3.06%). Other cancer types, including fallopian tubes and broad ligaments, gallbladder and extrahepatic bile ducts, kidney, bladder, prostate, oral cavity, oropharynx, and hypopharynx, among patients with vitamin D deficiency, were also presented.

**Table 2:** Cancer types of patients with vitamin D deficiency.

Cancer type	Number of patients with cancer (*N* = 229)	ICD-O-3
Colon, rectum, sigmoid junction and anus	32 (13.97)	C18–C21
Liver and intrahepatic bile ducts	31 (13.54)	C22
Breast	29 (12.66)	C50
Lungs, bronchi and trachea	25 (10.92)	C33–C34
Hematopoietic system (including lymphomas)	19 (8.29)	C42, C77
Thyroid	14 (6.11)	C73
Cervical, ovarian, and uterine	11 (4.80)	C53–C56
Stomach	10 (4.37)	C16
Skin	9 (3.93)	C44
Pancreatic	7 (3.06)	C25
Fallopian tubes and broad ligaments	6 (2.62)	C57
Gallbladder and extrahepatic bile ducts	6 (2.62)	C24
Kidney	5 (2.18)	C64
Bladder	4 (1.75)	C67
Prostate	4 (1.75)	C61
Oral cavity, oropharynx, and hypopharynx	3 (1.31)	C00–C14

The risk factors for new-onset cancer among patients with vitamin D deficiency are shown in Table 3. The crude HRs indicated that nearly all of the selected confounding factors, with the exception of sex, demonstrated significant effects on the risk of developing cancer. However, after considering all selected confounding factors, the results found that patients aged between 50 and 65 years and more than 65 years indicated a 3.10-fold (95% C.I.: 2.12–4.51; *p* < 0.0001) and 4.55-fold (95% C.I.: 3.03–6.82; *p* < 0.0001) cancer incidence rate, respectively compared with those younger than 50 years. Moreover, patients with comorbidities of DM (HR: 1.56; 95% C.I.: 1.01–2.41; *p* = 0.0463) presented a higher cancer incidence rate than those without DM. For those vitamin D deficiency patients with liver diseases, they show a significant 1.62-fold (95% C.I.: 1.03–2.54; *p* = 0.0384) risk of developing cancer compared with those without liver disease.

**Table 3:** Risk of cancer among vitamin D deficiency patients.

	Crude HR	*p*-value	AHR	*p*-value
Sex				
Male	Ref.		Ref.	
Female	1.02 (0.77–1.36)	0.8711	0.86 (0.65–1.15)	0.3104
Age group				
<50	Ref.		Ref.	
50–65	3.37 (2.34–4.84)	<0.0001	3.10 (2.12–4.51)	<0.0001
> = 65	5.45 (3.81–7.80)	<0.0001	4.55 (3.03–6.82)	<0.0001
CCI group				
0	Ref.		Ref.	
1–2	2.36 (1.78–3.14)	<0.0001	1.20 (0.81–1.80)	0.3645
> = 3	3.96 (2.67–5.86)	<0.0001	1.32 (0.62–2.84)	0.4733
Comorbidity				
CHF	2.89 (1.57–5.30)	0.0006	1.40 (0.73–2.68)	0.3191
CVA	1.77 (1.03–3.05)	0.0380	0.87 (0.48–1.59)	0.6568
Dementia	3.43 (1.61–7.30)	0.0014	1.61 (0.73–3.56)	0.2370
CPD	2.57 (1.71–3.87)	<0.0001	1.51 (0.94–2.41)	0.0869
Renal disease	2.36 (1.50–3.70)	0.0002	1.22 (0.68–2.21)	0.5086
Liver disease	2.32 (1.58–3.39)	<0.0001	1.62 (1.03–2.54)	0.0384
DM	2.64 (1.91–3.64)	<0.0001	1.56 (1.01–2.41)	0.0463
Hyperlipidemia	1.58 (1.12–2.22)	0.0093	0.79 (0.54–1.14)	0.2087
HTN	2.02 (1.53–2.65)	<0.0001	0.90 (0.65–1.23)	0.4962

AHR, adjusted hazard ratio; CHF, congestive heart failure, CVA, cerebrovascular disease, CPD, chronic pulmonary disease, DM, diabetes mellitus, HTN, hypertension.

**Figure 2:**
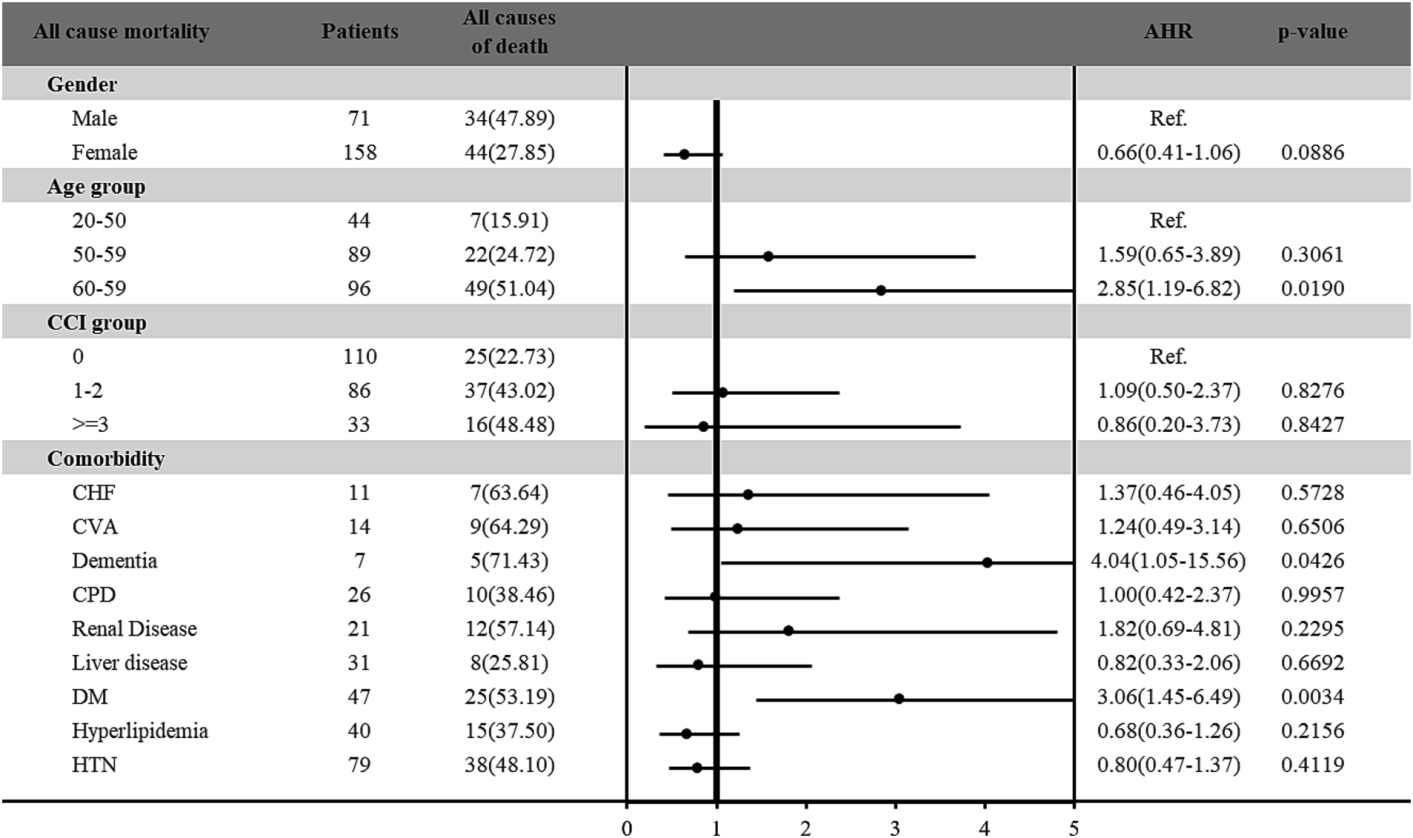
Risk of all causes mortality among vitamin D deficiency patients with cancer.

**Figure 3:**
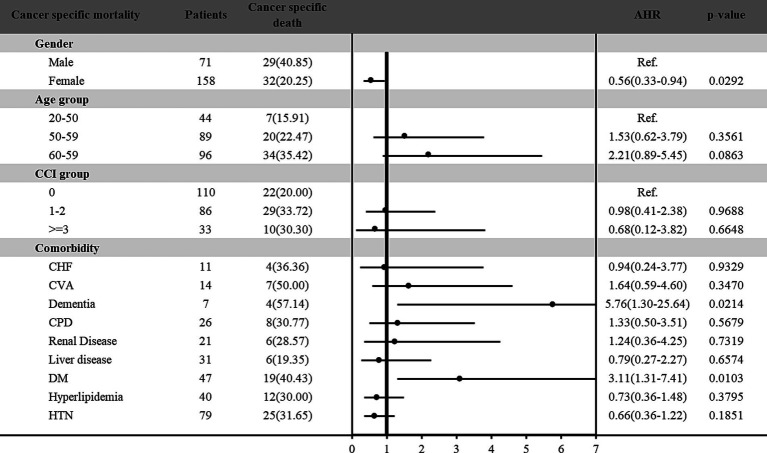
Risk of cancer-specific mortality among vitamin D deficiency patients with cancer.

Furthermore, the risk of all-cause and cancer-specific mortality among vitamin D deficiency patients with cancer was estimated in Figures 2, 3, respectively. Among the 229 patients with vitamin D deficiency and diagnosed with cancer, a total of 78 patients (34.1%) were all-cause deaths, and 61 patients (26.6%) had died due to cancer-related causes. Male patients and older patients (aged more than 65 years old) with vitamin D deficiency and cancer presented higher all-cause and cancer-specific mortality rates. Female patients with vitamin D deficiency and cancer presented a lower cancer-specific mortality risk (HR: 0.56; 95% C.I.: 0.33–0.94; *p* = 0.0292) than male patients. In different age groups, patients aged more than 65 presented significantly higher all-cause mortality risk (HR: 2.85; 95% C.I.: 1.19–6.82; *p* = 0.0190) compared with younger patients (age < 50). In addition, patients with DM had 3.06-fold (95% C.I.: 1.45–6.49; *p* = 0.0034) all-cause mortality and 3.11-fold (95% C.I.: 1.31–7.41; *p* = 0.0103) cancer-specific mortality among vitamin D deficiency patients with cancer than those without DM. Furthermore, our results found that vitamin D deficiency patients with a history of cancer and dementia also indicated a significantly higher all-cause (HR: 4.04; 95% C.I.: 1.05–15.56; *p* = 0.0426) and cancer-specific (HR: 5.76; 95% C.I.: 1.30–25.64; *p* = 0.0214) mortality risk than those without dementia.

## 4 Discussion

Studies have shown that vitamin D deficiency is associated with increased cancer risks (11, 16, 22). However, the results of existing large RCTs and meta-analyses on the benefit of unscreened vitamin D supplementation on cancer incidence and mortality remain controversial (12, 13, 19, 23, 24). Therefore, identifying populations with certain characteristics that suggest a potentially high sensitivity to vitamin D supplementation becomes critically important (25). We found that CRC cancer was the most prevalent cancer type in our cohort, followed by liver, breast, and lung cancer. Patients with older age, liver diseases, and DM were associated with higher cancer incidence, while DM was associated with higher mortality among cancer patients.

### 4.1 Types of cancer in vitamin D-deficient patients

Previous literature provided a strong consensus for an inverse relationship between circulating 25(OH)D and colorectal cancer (CRC) incidence (26–28) and mortality (29, 30). CRC is also the leading cancer type among our cohort. A recent meta-analysis provided evidence that a higher concentration of circulating vitamin D conferred protection against liver cancer (31). Taiwan is an endemic area of viral hepatitis and thus has a high incidence of chronic liver disease and hepatocellular carcinoma (HCC), which may explain why liver cancer is the second most common cancer in our study (32, 33). Furthermore, the inverse association between the circulating concentration of 25(OH)D and the risk of breast cancer and lung cancer was also documented in previous review articles, but the effect of vitamin D supplementation on the prevention of these cancer types has not been well investigated (34, 35).

Vitamin D and its metabolites have demonstrated anti-cancer activity through various mechanisms. These include inhibiting tumor angiogenesis (2, 3), suppressing clonal proliferation (4, 5), and promoting cell differentiation and apoptosis (6, 7). Additionally, vitamin D enhances the DNA repair process and autophagy in cancer cells (36). It also hinders tumorigenesis through its anti-inflammatory properties, which involve reducing the expression of pro-inflammatory cytokines and regulating inflammatory signaling pathways. Furthermore, vitamin D exerts antioxidative effects by modulating the expression of genes related to antioxidants (36). These mechanisms collectively underscore the potential role of vitamin D in cancer prevention and support the importance of further investigation, particularly in the context of supplementation.

### 4.2 Determinants of cancer incidence among people with vitamin D deficiency

Identifying risk factors for cancer incidence in vitamin D-deficient populations is a key step in individualized health education and targeted supplementation, since many meta-analyses have shown that unscreened vitamin D supplementation does not reduce the cancer incidence rate (13, 14, 19).

Diabetic patients were found to have an increased risk of several types of cancer, including colon rectum, breast, liver, and prostate (37). Current review articles further propose that vitamin D deficiency may be one of the important factors responsible for increased cancer risk among diabetic patients (36). Previous studies also indicated vitamin D can reduce several diabetes-driven cancer risk factors, such as hyperglycemia, insulin resistance, inflammation, and oxidative stress (36, 38, 39). Unfortunately, patients with DM more frequently suffer from vitamin D deficiency (40). Targeted supplementation in this population may have the potential benefit of cancer prevention and glycemic control (40).

Vitamin D deficiency is highly prevalent among patients with chronic liver disease, and it is associated with disease progression, increasing liver fibrosis severity and mortality (41, 42). A recent systemic review and meta-analysis also demonstrated a reverse relationship between the concentration of circulating vitamin D and liver cancer risk (31). The possible underlying mechanisms included the anti-inflammatory role of vitamin D, which reduced oxidative stress and hepatocarcinogenesis (43, 44), the downregulation of the expression of tumor growth factorβ (TGFβ) (44), and the inhibition of neoangiogenesis mediated by vascular endothelial growth factor (VEGF) (2). The specific domestic condition and the significant adverse impact of vitamin D deficiency on the cancer risk among this population revealed in this study may suggest a prospective benefit of vitamin D supplementation, and further large-scale studies for evaluation are warranted.

### 4.3 Determinants of mortality among cancer patients with vitamin D deficiency

Our result revealed that older age, dementia, and diabetes were significant risk factors for death among cancer patients with vitamin D deficiency. Older age is a risk factor for low levels of vitamin D because both the nutritional intake and cutaneous synthesis of vitamin D decrease with aging (45). The DO-HEALTH trial, a large multicenter RCT conducted in five European countries, demonstrated that the supplementation of vitamin D3 plus omega-3 s and simple exercise significantly reduced cancer risk in adults ≥70 years (adjusted HR 0.39, 95% CI 0.18–0.85) (46). Furthermore, it has been estimated that vitamin D supplementation in Germany could prevent approximately 30,000 cancer-related deaths annually and may have the potential to save costs (47). Our study indicated that patients with vitamin D deficiency had a significantly increased risk of cancer incidence among those with older age, liver disease, and DM. In addition, patients with vitamin D deficiency and cancer had a higher mortality risk than older patients. Targeted vitamin D supplementation in the older population may be an effective and economical approach to alleviate the burden of cancer and other age-related diseases.

The relationship between dementia and cancer is complex. Preexisting dementia has been documented to be associated with poorer outcomes and increased mortality in cancer patients (48). This may be due to several factors, including older age (49), more advanced stages of cancer at diagnosis (50), and a reduced likelihood of receiving curative treatments such as surgery or chemotherapy (51). Low vitamin D concentrations in older adults have been linked to neurodegeneration and an increased risk of cognitive decline in previous studies (52, 53). Vitamin D and its metabolites are believed to influence cognitive function through several potential mechanisms. First, they play a role in the regulation of neural cell development and the maintenance of proper neural function (54). Additionally, vitamin D has been associated with the stimulation of neurotrophic agents, such as glial-derived neurotrophic factor (GDNF) and nerve growth factor (NGF) (55). These agents are responsible for promoting the growth and maintenance of neurons in the nervous system. Moreover, vitamin D exhibits a capacity to protect neurons from cytotoxicity and apoptosis, which can be induced by factors such as amyloid-β (Aβ) (56). Aβ is associated with neurodegenerative processes.

The deterioration of cognitive function can have significant consequences, including poor symptom recognition and potential delays in the diagnosis of various health conditions, such as cancer. This delay in diagnosis may contribute to an increase in mortality rates, affecting both cancer and non-cancer causes of mortality (48, 50). Our study found that the presence of dementia was associated with a significant risk of mortality among cancer patients with vitamin D insufficiency. However, further prospective studies are needed to explore the effect of vitamin D supplementation on recognition preservation and survival in patients with dementia, as previous studies have yielded conflicting results (57, 58).

Studies investigating the relationship between vitamin D concentration and cancer mortality among diabetic patients were limited. A retrospective cohort study conducted by Wong et al. found that diabetes had an insignificant trend toward increasing cancer mortality among older women (59). The relatively smaller sample size and different study populations may have contributed to the statistically insignificant results in this study.

### 4.4 Strengths and limitations

The main strength of the current study is that it used a nationwide population-based database, which provides comprehensive and representative data, to investigate cancer incidence and mortality among patients with vitamin D deficiency. According to the current findings, future research can link these epidemiological parameters from the real-world database with specific biochemical parameters. Therefore, researchers may gain deeper insights into the potential mechanisms between patients with vitamin D deficiency and cancer to establish valid strategies for prevention and treatment.

However, there are several limitations that need to be noted. First of all, the diagnosis of vitamin D deficiency in the current study was defined using the ICD code from the NHIRD. No sub-group analysis was conducted in this study to explore dose–response patterns based on increments of circulating serum 25(OH)D levels. Therefore, future research could focus on investigating the potential dose–response relationship between serum 25(OH)D levels and the development of cancer. Second, the blood samples were not necessarily drawn near the time of cancer diagnosis, which may have some influence on the results of the association between vitamin D deficiency and cancer risk (60). However, people who had cancer before the diagnosis of vitamin D deficiency were excluded from our study. Third, the NHIRD lacks comprehensive patient information for adjustment, such as tobacco and alcohol use status, family history of cancer, and body mass index, which may also have an impact on the incidence and mortality of cancer and affect the research results. Finally, the sub-group analysis of the clinical stages of different cancer types upon diagnosis and treatment methods was not conducted in this study.

## 5 Conclusion

Due to inconclusive findings in the existing literature on the potential benefits of unscreened vitamin D supplementation in reducing cancer risk, it is crucial to identify populations that may benefit from targeted vitamin D supplementation. Our study revealed that vitamin D-deficient individuals with liver disease had an increased incidence of cancer, while those with dementia had an increased mortality rate among cancer patients. Additionally, older age and diabetes mellitus were associated with both increased cancer incidence and mortality. Therefore, targeted vitamin D supplementation in these populations may represent an effective and cost-efficient approach to reducing the burden of cancer and promoting overall health. Further large-scale prospective studies are warranted to investigate the actual impact of such interventions.

## Data availability statement

The datasets presented in this article are not readily available because the data sources are the Taiwan Nation Health Insurance Database and Taiwan Cancer Registry. Restrictions apply to the availability of these data, which were used under license for this study. Requests to access the datasets should be directed to the data are available with permission from the Taiwan Health and Welfare Data Science Center (https://dep.mohw.gov.tw/DOS/cp-5119-59201-113.html, Accessed on July 9, 2023).

## Ethics statement

The studies involving humans were approved by Ethics Committee of the Institutional Review Board of Chi-Mei Hospital. The studies were conducted in accordance with the local legislation and institutional requirements. Written informed consent for participation was not required from the participants or the participants’ legal guardians/next of kin in accordance with the national legislation and institutional requirements.

## Author contributions

Y-CL: Conceptualization, Methodology, Writing – original draft. Y-HC: Conceptualization, Methodology, Writing – original draft. F-WL: Methodology, Validation, Writing – review & editing. Y-CW: Methodology, Visualization, Writing – original draft. J-JW: Supervision, Writing – review & editing. S-WL: Validation, Writing – review & editing. C-HH: Conceptualization, Formal analysis, Methodology, Writing – original draft, Writing – review & editing, Validation.
